# Decoding the Ubiquitin-Mediated Pathway of Arthropod Disease Vectors

**DOI:** 10.1371/journal.pone.0078077

**Published:** 2013-10-21

**Authors:** Anthony Choy, Maiara S. Severo, Ruobai Sun, Thomas Girke, Joseph J. Gillespie, Joao H. F. Pedra

**Affiliations:** 1 Institute for Integrative Genome Biology, Center for Disease Vector Research and Department of Entomology, University of California Riverside, Riverside, California, United States of America; 2 "Institute for Integrative Genome Biology, Center for Plant Cell Biology, Department of Botany and Plant Sciences, University of California Riverside, Riverside, California, United States of America; 3 Department of Microbiology and Immunology, University of Maryland School of Medicine, Baltimore, Maryland, United States of America; Kansas State University, United States of America

## Abstract

Protein regulation by ubiquitin has been extensively described in model organisms. However, characterization of the ubiquitin machinery in disease vectors remains mostly unknown. This fundamental gap in knowledge presents a concern because new therapeutics are needed to control vector-borne diseases, and targeting the ubiquitin machinery as a means for disease intervention has been already adopted in the clinic. In this study, we employed a bioinformatics approach to uncover the ubiquitin-mediated pathway in the genomes of *Anopheles gambiae, Aedes aegypti, Culex quinquefasciatus, Ixodes scapularis, Pediculus humanus* and *Rhodnius prolixus*. We observed that (1) disease vectors encode a lower percentage of ubiquitin-related genes when compared to *Drosophila melanogaster*, *Mus musculus* and *Homo sapiens* but not *Saccharomyces cerevisiae*; (2) overall, there are more proteins categorized as E3 ubiquitin ligases when compared to E2-conjugating or E1-activating enzymes; (3) the ubiquitin machinery within the three mosquito genomes is highly similar; (4) ubiquitin genes are more than doubled in the Chagas disease vector (*R. prolixus*) when compared to other arthropod vectors; (5) the deer tick *I. scapularis* and the body louse (*P. humanus*) genomes carry low numbers of E1-activating enzymes and HECT-type E3 ubiquitin ligases; (6) *R. prolixus* have low numbers of RING-type E3 ubiquitin ligases; and (7) *C. quinquefasciatus* present elevated numbers of predicted F-box E3 ubiquitin ligases, JAB and UCH deubiquitinases. Taken together, these findings provide novel opportunities to study the interaction between a pathogen and an arthropod vector.

## Introduction

Vector-borne diseases are some of the most prevalent infectious illnesses worldwide. According to the World Health Organization, there are approximately 216 million cases of malaria alone, with over 1.2 million estimated deaths [1]. Dengue fever, another prominent vector-borne disease is responsible for 50-100 million cases each year, and it is making one of the fastest growing infectious maladies. An estimated 120 million individuals are affected each year by lymphatic filariasis [2]. Lyme disease, the most common tick-borne illness in the northern hemisphere, is responsible for 30,000 clinical cases in the United States alone with the actual number possibly being much higher [3]. Overall, vector-borne diseases are responsible for 16% of disability-adjusted life years [4], and these statistics are compounded by the social costs that also affect heavily-infected communities.

Ubiquitin consists of a 76 amino acid protein that carries seven lysine (K) residues (K6, K11, K27, K29, K33, K48 and K63) [5]. Ubiquitin contributes to several fundamental biological processes within cells including, but not limited to: protein turnover, endocytosis, DNA repair, transcription and immunity [6,7]. Ubiquitination involves a ubiquitin-activating enzyme (E1), a ubiquitin-conjugating enzyme (E2) and a ubiquitin-protein ligase (E3) [5]. Ubiquitination is counteracted by de-ubiquitination, and de-ubiquitinases remove ubiquitin chains from molecules by cleaving precursors enzymatically, eliminating ubiquitin from proteins, or by altering the ubiquitin linkage type [5]. 

In *Drosophila*, it is established that developmental regulation by the evolutionarily conserved Notch receptor depends on ubiquitin [8], and some proteins associated with neuronal control and neurodegenerative disorders undergo ubiquitination [9]. Additionally, there is abundant evidence showing that ubiquitination licenses the Toll, the janus kinase (JAK)/signal transducer and activator of transcription (STAT) and the immunodeficiency (IMD) pathways during immune challenge against bacterial, viral and fungal infection [5]. This immunological circuitry is not unique to *Drosophila* because ubiquitination also regulates these pathways in disease vectors, such as: *Anopheles gambiae*, *Aedes aegypti*, *Culex quinquefasciatus* and *Ixodes scapularis* [5].

The lack of information regarding the role of ubiquitin in disease vectors is problematic since molecular interactions depend on this posttranslational modification. These biochemical signatures may lead to novel factors to control pathogen transmission and/or acquisition [5]. For instance, drugs targeting the ubiquitin machinery for therapeutic development have entered clinical trials (http://www.clinicaltrials.gov) and have been approved by the Food and Drug Administration for use (e.g., Bortezomib - Millennium Pharmaceuticals and Nutlin - Roche) [5]. In this study, we used computational biology to identify the ubiquitin machinery of six clinically-relevant arthropod vectors: *An. gambiae, Ae. aegypti, C. quinquefasciatus, I. scapularis, Pediculus humanus* and *Rhodnius prolixus*. We compared our findings to datasets available for *Saccharomyces cerevisiae*, *Drosophila melanogaster*, *Mus musculus* and *Homo sapiens* [10-13]. While computational biology has been extensively used as a methodology in disease-causing arthropods for comparative genomics [14,15], transcriptomics [14,16-19] and quantitative proteomics [14,20,21], it has not been fully utilized to uncover the machinery associated with posttranslational modifications. 

## Materials and Methods

### Protein and domain datasets

Complete protein datasets encoded within the genomes of *I. scapularis, An. gambiae, Ae. aegypti, C. quinquefasciatus, P. humanus* and *R. prolixus* were downloaded from VectorBase [22]. Protein datasets from *S. cerevisiae, D. melanogaster, H. sapiens* and *M. musculus* were downloaded from http://www.yeastgenome.org [23], http://www.flybase.net [24], and http://ncbi.nih.gov [25], respectively (Table S1). Based on a previously published study twenty-five Pfam domains for the ubiquitination machinery were selected [13], and downloaded from the Hidden Markov Model (HMM) library version 26.0 [26]. This included the following Pfam domains: APG12, PF04110; Atg8, PF02991; ubiquitin, PF00240; Ufm1, PF03671; Urm1, PF09138; ThiF, PF00899; UBACT, PF02134; UQ-con, PF00179; zf-C3HC4, PF00097; zf-Apc11, PF12861; RINGv, PF12906; Rtf2, PF04641; HECT, PF00632; Cullin, PF00888; U-box, PF04564; F-box, PF00646; OTU, PF02338; Josephin, PF02099; JAB, PF01398; DUF862, PF05903; WLM, PF08325; UCH, PF00443; Peptidase_C12, PF01088; Peptidase_C48, PF02902; and Peptidase_C54, PF03416.

### Protein identification

Using the selected Pfam domains, the hmmsearch program of the HMMER v3.0 package [27] was employed to identify analogous proteins within the chosen species (Tables S2, S3, S4, S5, S6, S7, S8, S9, S10). Proteins were assigned to the corresponding Pfam domains if searches returned an E-value <0.5. Importantly, observed discrepancies between our results and findings previously published for *S. cerevisiae, D. melanogaster, H. sapiens* and *M. musculus* [10-13] were determined to result from one or more of the following: (1) changes made in the databases; (2) different programs and search parameters employed across studies; and (3) the high sensitivity of the relatively new HMMER 3.0 algorithm in detecting remotely related sequence similarities. 

### Computation of multiple alignments

 The resulting protein sequences from *I. scapularis, An.gambiae, Ae. aegypti, and C. quinquefasciatus* were aligned using the Multiple Sequence Alignment tool MUSCLE [23] available on the Phylogeny.fr site [24]. The full mode option was utilized for the MUSCLE program and contained 3 stages: 1) draft progressive alignment, 2) improved progressive alignment, and 3) alignment refinement. Maximum number of iterations was 16, the default option. We did not remove any poorly aligned regions prior to phylogeny estimation because we were primarily interested in the relationship of proteins. 

### Generation of protein phylogenies

 For each aligned dataset, protein phylogenies were estimated with the maximum likelihood method, employing the PhyML program [28] available through Phylogeny.fr. We presented cladograms, as opposed to phylograms to maintain readability of the large numbers of sequences considered in this study. Cladrograms are also sufficient for illustrating the underlying function of related proteins, rather than inferring evolutionary relationships. 

### Identification of subfamilies/clusters

Protein sequences belonging to specific subcategories of the five ubiquitination-components (ubiquitin/ubiquitin-like proteins, ubiquitin-activating enzymes (E1), ubiquitin-conjugating enzymes (E2), ubiquitin ligases (E3) and deubiquitinases) were identified based on the Pfam domain matches obtained from hmmsearch. Whenever possible, proteins were cataloged according to their major branching patterns in their respective phylogenies: ubiquitin/ubiquitin-like proteins (UB S30, small ubiquitin**-**like modifier (SUMO), neural precursor cell expressed developmentally downregulated gene 8 (NEDD8), autophagy-related protein 8 (ATG8), ubiquitin-related modifier-1 (URM1), homologous to ubiquitin 1 (HUB1), E1s (ubiquitin-activating enzyme 1 (UBA1), UBA2, UBA3, UBA1-like, autophagy-related E1-like enzyme (ATG7)), really interesting new gene (RING) (zf-C3HC4, zf-Apc11, RINGv, Rtf2), other E3s (homology to E6AP C-terminus (HECT), Cullin, U-box), and DUBs (ovarian tumor (OTU), Josephin, jun activation domain-binding protein (JAB), domain of unknown function (DUF) 862, Wss1p‐like metalloproteases (WLM), ubiquitin carboxyl-terminal hydrolase (UCH), Peptidase_C12, Peptidase_C48, and Peptidase_C54). For direct classification of ubiquitin-like proteins, we manually analyzed and categorized each protein encoded in the genomes of *An. gambiae, Ae. aegypti, C. quinquefasciatus*, and *I. scapularis*. Ubiquitin-like proteins analyzed were SUMO, URM1, NEDD8, HUB1 and ATG8. Proteins containing two or more Pfam domains were observed and recorded in Table S11. Bootstrap values were used to evaluate the consistency of the cluster subcategories.

## Results and Discussion

### Composition of the ubiquitin machinery in model organisms versus disease vectors

**Figure 1:**
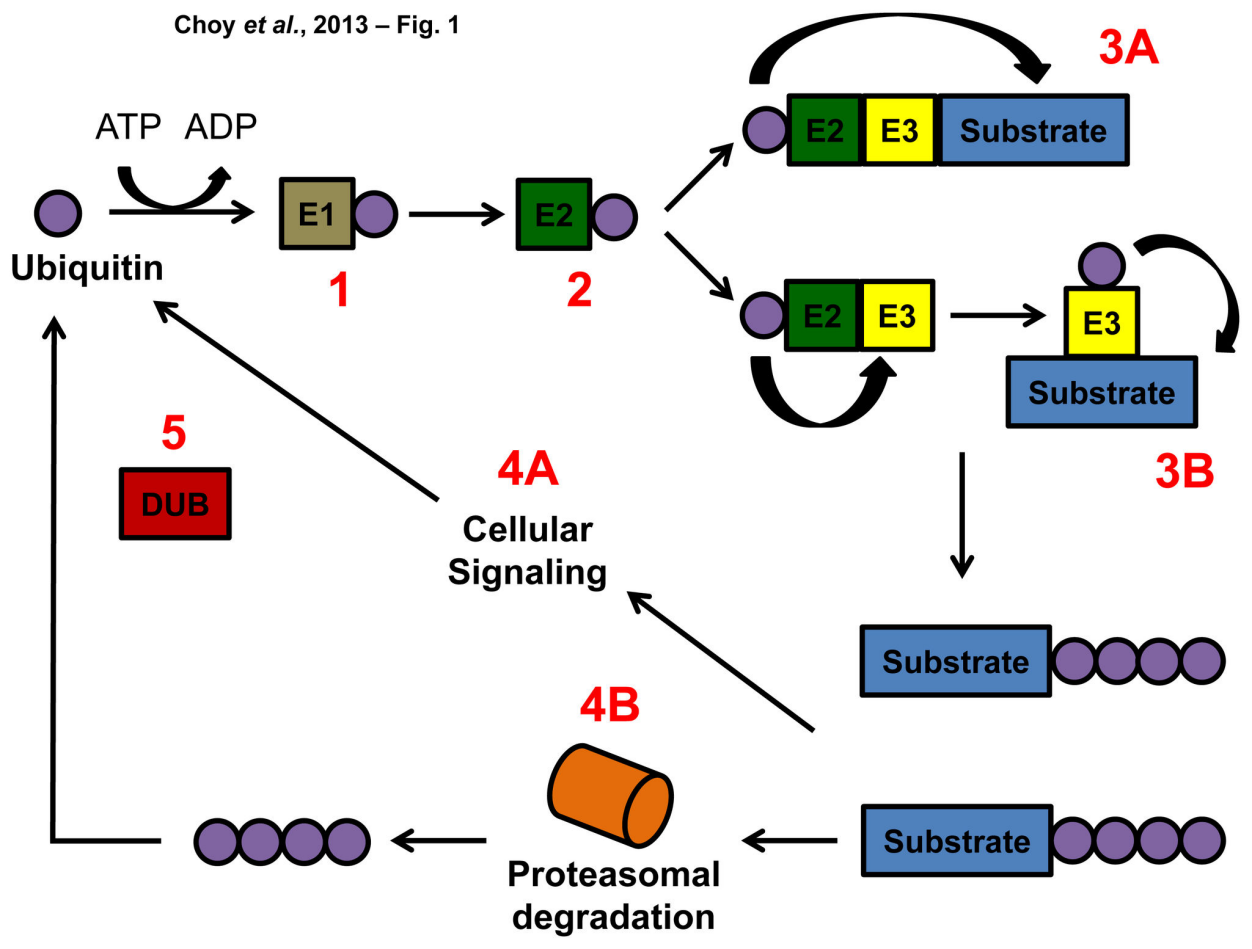
Schematic of the ubiquitination pathway. (1) A thiol ester bond between the E1-activating enzyme and ubiquitin is formed during ubiquitin activation. (2) The activated ubiquitin is then transferred to the E2-conjugating enzyme. (3) How ubiquitin is added onto a substrate varies according to the ubiquitin ligase (E3) category. (3A) RING-type E3 ligases function as a molecular scaffold that position the E2-conjugating enzyme with the substrate. (3B) HECT-type E3 ligases form an intermediary complex with ubiquitin leading to its transfer from the E2-conjugating enzyme to the substrate. (4A) Polyubiquitinated substrates may be targeted for cellular signaling or (4B) proteasomal degradation depending on the ubiquitin linkages (5). De-ubiquitinases (DUBs) recycle ubiquitin completing the cycle.

**Figure 2:**
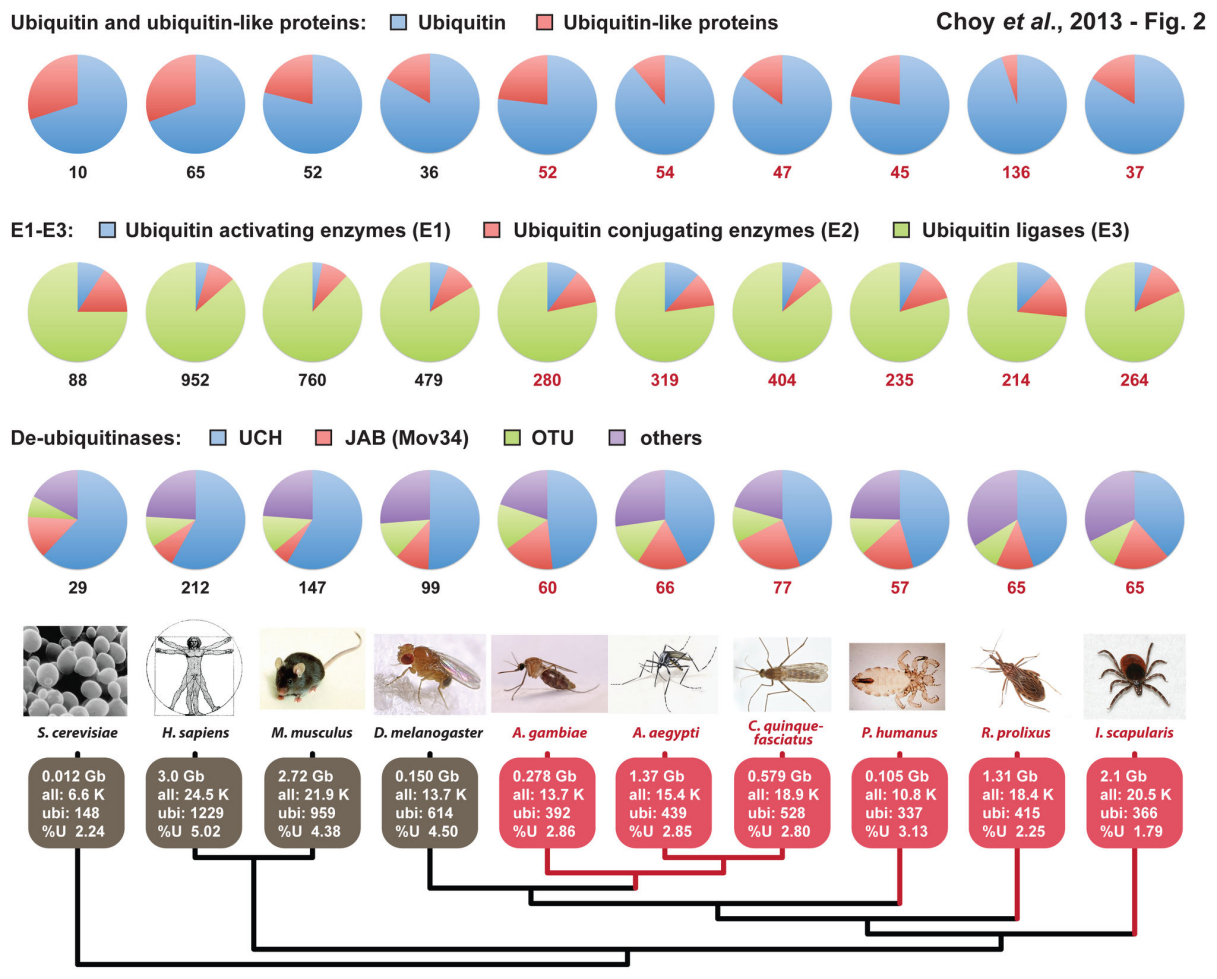
Proteins encoded for the ubiquitin machinery within the genomes of four model eukaryotes and six medically important arthropod vectors. Ten species are depicted at bottom, with model organisms colored gray (*S. cerevisiae*, *H. sapiens*, *M. musculus* and *D. melanogaster*) and arthropod vectors colored red (*A. gambiae*, *A. aegypti*, *C. quinquefasciatus*, *P. humanus*, *R. prolixus* and *I. scapularis*). The statistics shown are genome size in giga base pairs (Gb) and “all” referring to total number of predicted genes. “Ubi” refers to the total number of characterized (model organisms) or predicted (vectors) ubiquitin machinery proteins encoded within each genome, with “%U” the proportion of ubiquitin-related genes. Proteins were categorized into three major groups: Ubiquitin and ubiquitin-like proteins, E1-E3 enzymes, and de-ubiquitinases. For each genome, a breakdown within each major group depicts the composition of protein families (sub-groups).

 The ubiquitin machinery regulates fundamental biological processes within eukaryotic cells (Figure 1). Thus, we used a bioinformatics approach to characterize this pathway in arthropod disease vectors. Our analysis demonstrated that the genomes of three clinically-relevant mosquitoes carried a similar ubiquitin pathway to total gene ratio (%U), ranging from 2.80 to 2.86 (Figure 2). *I. scapularis* contained the smallest %U (1.79), while *P. humanus* had the highest ratio at 3.13. *R. prolixus* had a %U of 2.25. When compared to four model species, all disease vectors possessed a much lower %U, with the model species ranging from 4.38 to 5.02. The exception was *S. cerevisiae* (%U = 2.24). Interestingly, *D. melanogaster* carried a much higher %U at 4.50. 

### Ubiquitin

**Figure 3:**
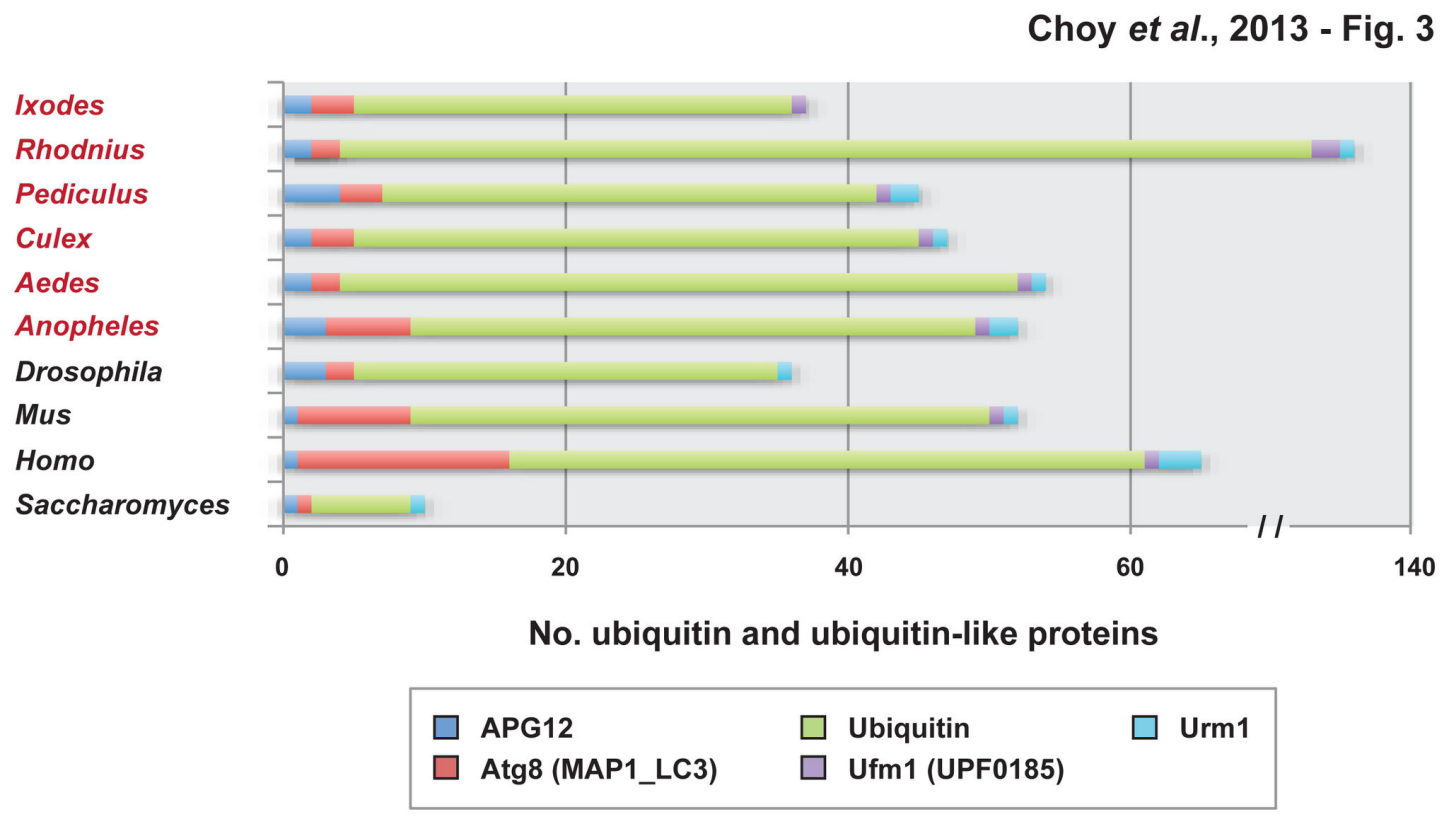
Composition of ubiquitin and ubiquitin-like proteins within the genomes of four model eukaryotes and six medically important arthropod vectors. Using HMMER v.3.0, complete sets of proteins for all ten genomes were scanned for the presence of five Pfam domain models for ubiquitin and ubiquitin-like proteins: APG12 (PF04110), Atg8 (PF02991), ubiquitin (PF00240), Ufm1 (PF03671), and Urm1 (PF09138). Only protein matches to Pfam models with E-values <0.05 were selected for compilations.

 Using the ubiquitin Pfam domain, we analyzed the number of ubiquitin molecules reported for each of the selected species: *I. scapularis* (31), *An.gambiae* (40), *Ae. aegypti* (48), *C. quinquefasciatus* (40), *P. humanus* (35) and *R. prolixus* (129) (Table 1; Figure 3). We also analyzed the ubiquitin number in *S. cerevisiae* (7), *D. melanogaster* (30), *H. sapiens* (45) and M. musculus (41). Apart from *S. cerevisiae* (7) and *R. prolixus* (129), our analysis revealed a similar number of ubiquitin proteins across species. We further analyzed sequence similarities among ubiquitin family members (e.g, ubiquitin and ubiquitin-like proteins) for *An. gambiae, Ae. aegypti, C. quinquefasciatus*, and *I. scapularis* from their multiple alignments (Figure S1). 

**Table 1:** In silico ubiquitin machinery of medically relevant arthropod vectors when compared to known protein datasets* .

**Domains/Genomics**	***I. scapularis***	***An. gambiae***	***Ae. aegypti***	***C. quinquefasciatus***	***P. humanus***	***R. prolixus***	***S. cerevisiae***	***D. melanogaster***	***H. sapiens***	***M. musculus***
**Ubiquitin and ubiquitin-like proteins**										
**APG12**	2	3	2	2	4	2	1	3	1	1
**Atg8 (MAP1_LC3)**	3	6	2	3	3	2	1	2	15	8
**Ubiquitin**	31	40	48	40	35	129	7	30	45	41
**Ufm1 (UPF0185)**	1	1	1	1	1	2	0	0	1	1
**Urm1**	0	2	1	1	2	1	1	1	3	1
										
**Ubiquitin activating enzymes (E1)**										
**ThiF/UBACT**	16	29	37	30	19	26	8	30	42	25
										
**Ubiquitin conjugating enzymes (E2)**										
**UQ_con**	32	32	36	28	29	31	14	49	87	67
										
**Ubiquitin ligases (E3)**										
**RING**	151	147	161	156	127	86	43	309	631	501
**HECT**	9	17	17	23	14	16	5	20	45	34
**Cullin**	5	6	9	4	7	8	4	14	16	11
**U-Box**	8	10	10	12	7	8	2	11	20	18
**F-Box**	43	39	49	151	32	39	12	46	111	104
										
**De-ubiquitinases**										
**OTU**	7	9	9	9	7	6	2	12	21	18
**Josephin**	2	1	1	2	2	2	0	1	13	10
**JAB (Mov34)**	12	10	11	18	10	8	4	11	17	8
**DUF862**	3	1	2	3	2	11	0	5	8	2
**WLM**	2	2	0	1	0	0	1	2	0	0
**UCH**	25	29	28	34	26	29	18	50	123	86
**Peptidase_C12**	4	2	7	3	4	3	1	6	7	6
**Peptidase_C48**	8	3	4	3	5	4	2	10	16	12
**Peptidase_C54**	2	3	4	4	1	2	1	2	7	5
**Total**	**366**	**392**	**439**	**528**	**337**	**415**	**127**	**614**	**1229**	**959**

Protein datasets were downloaded from online databases and matched with selected Pfam domains using the search program of the HMMER 3.0 package. Search results are listed individually.

### Ubiquitin-like proteins

 Ubiquitin-like proteins were first discovered in 1979 and named after the interferon-stimulated gene 15 (ISG15) [29]. Since its discovery, several other ubiquitin-like proteins have been identified. In our study, we analyzed the paralogs for several ubiquitin-like proteins (Table 1; Figure 3). Specifically, we used Pfam hmmsearch domains for APG12, ATG8, UFM1 and URM1. For direct classification of ubiquitin-like proteins, we manually analyzed and categorized each protein encoded in the genomes of *An. gambiae, Ae. aegypti, C. quinquefasciatus*, and *I. scapularis* (Figure S1). Ubiquitin-like proteins analyzed were SUMO, NEDD8, URM1, HUB1 and ATG8. 

 One of the most extensively studied ubiquitin-like proteins is SUMO, a polypeptide with around 100 residues and 18% identity to ubiquitin [30]. SUMO modification has been known to regulate a multitude of biological functions. Among the most prominent are protein localization, gene expression, and DNA repair [31]. We observed a total of six proteins under the description of SUMO: *An. gambiae* (2), *Ae. aegypti* (2), *C. quinquefasciatus* (1), and *I. scapularis* (1) (Figure S1). NEDD8 is an ubiquitin-like protein with 81 amino acid residues [32]. It is around 60% identical to ubiquitin. Previous reports have identified the ligation of NEDD8 to members of the cullin family protein, suggesting a critical role in cullin-RING E3 ubiquitin ligase (CRL) activation [33]. We observed four proteins categorized under the NEDD8 hmmsearch description for *An. gambiae, Ae. aegypti, C. quinquefasciatus* and *I. scapularis* - one from each vector (Figure S1).

URM1 is a ubiquitin-like protein that shares very little homology with ubiquitin [34]. Four proteins were categorized under the URM1 description for *An. gambiae, Ae. aegypti, C. quinquefasciatus*, and *I. scapularis* (Table 1). HUB1 differs from ubiquitin in that instead of a C-terminal tail with a GG motif, it carries a double tyrosine (YY) motif [35]. In our analysis, we reported four HUB1 proteins within *An. gambiae, Ae. aegypti, C. quinquefasciatus* and *I. scapularis*, with one being identified in each vector (Figure S1). ATG8 is one of the most extensively studied ubiquitin-like proteins and has been identified as being a key component in the regulation of autophagy [36]. Of the ubiquitin-like proteins examined, the number of proteins similar to ATG8 was ranked the highest: *An. gambiae* (6), *Ae. aegypti* (2), *C. quinquefasciatus* (3), and *I. scapularis* (3) (Table 1). 

### Ubiquitin-activating enzymes (E1)

**Figure 4:**
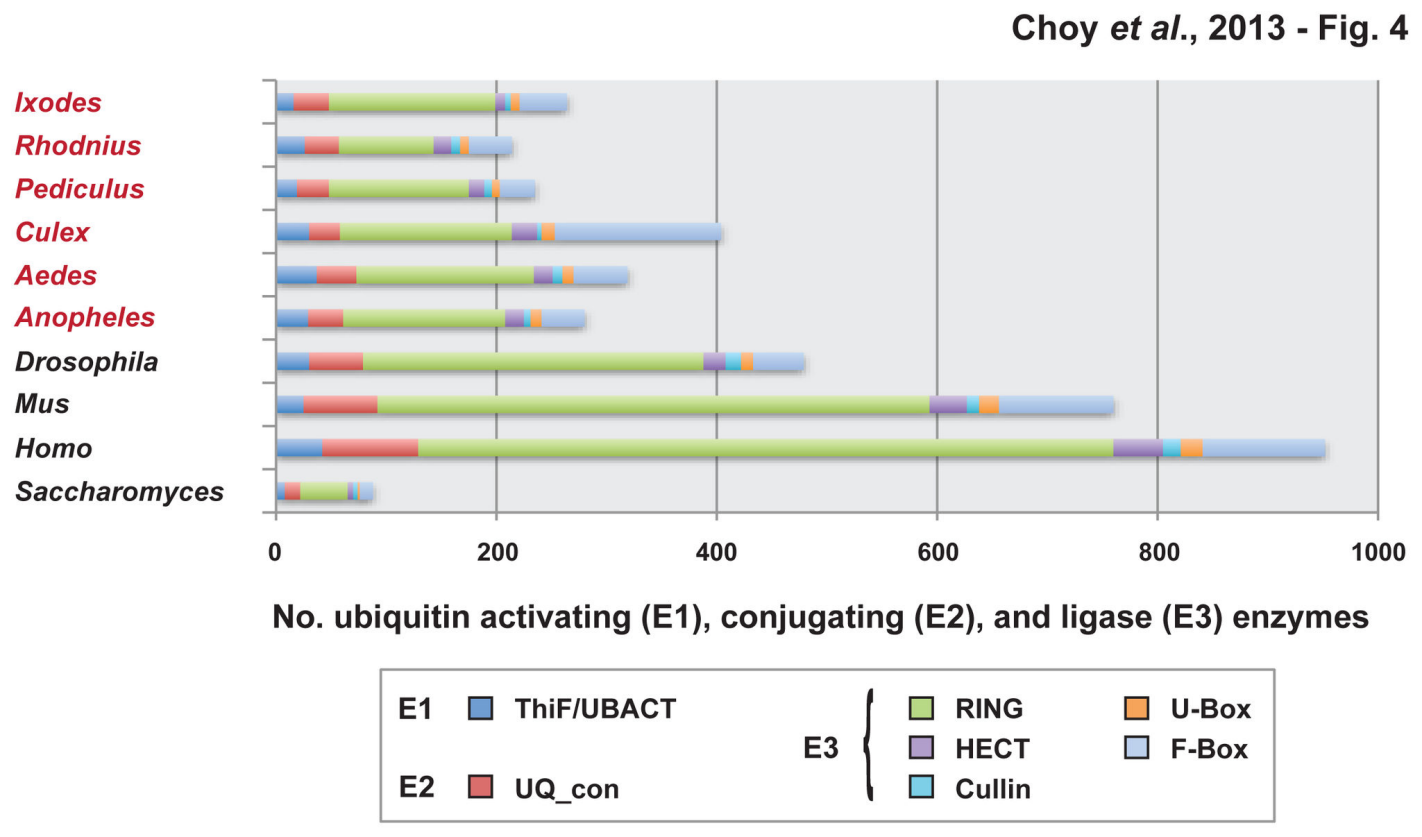
Composition of ubiquitin-activating enzymes (E1), ubiquitin conjugating enzymes (E2), and ubiquitin ligases (E3) within the genomes of four model eukaryotes and six medically important arthropod vectors. Using HMMER v.3.0, complete sets of proteins for all ten genomes were scanned for the presence of two Pfam domain models for ubiquitin activating enzymes (E1): ThiF (PF00899) and UBACT (PF02134); one Pfam domain model for ubiquitin conjugating enzymes (E2): UQ-con (PF00179); and eight Pfam domain models for ubiquitin ligases (E3): zf-C3HC4 (PF00097), zf-Apc11 (PF12861), RINGv (PF12906), Rtf2 (PF04641), HECT (PF00632), Cullin (PF00888), U-box (PF04564), and F-box (PF00646). Only protein matches to Pfam models with E-values <0.05 were selected for compilations.

 There are several classes of ubiquitin-activating enzymes, distinguishable by their ubiquitin-activating (UBA) domain. The E1 associated to ubiquitin is known as UBA1. UBA2 and UBA3 serve as activators for SUMO and NEDD8 respectively [13]. The E1 for URM1 is UBA4 [37]. ATG7, the E1 associated to ATG8, is recognized by an N-terminal motif [38]. UBA1-like, is slightly larger than UBA1 [13]. Using its Pfam domain, we analyzed the number of E1 molecules reported for each of the selected species: *I. scapularis* (16), *An. gambiae* (29), *Ae. aegypti* (37), *C. quinquefasciatus* (30), *P. humanus* (19) and *R. prolixus* (26) (Table 1; Figure 4). The three mosquitoes (*An. gambiae*, *Ae. aegypti*, and *C. quinquefasciatus*) have a very similar number of E1 proteins, while *I. scapularis* and *P. humanus* have noticeably less E1s than the other disease vectors. We also analyzed the E1 number in *S. cerevisiae* (8), *D. melanogaster* (30), *H. sapiens* (42), and M. musculus (25) (Table 1). We further analyzed the proteins identified as E1 according to their subcategories for *An. gambiae, Ae. aegypti, C. quinquefasciatus* and *I. scapularis* through phylogeny estimation (Figure S2). 

### Ubiquitin-conjugating enzymes (E2)

 E2s consists of a conserved domain with approximately 150 amino acids [39]. We analyzed the number of E2 molecules reported for *I. scapularis* (32), *An. gambiae* (32), *Ae. aegypti* (36), *C. quinquefasciatus* (28), *P. humanus* (29) and *R. prolixus* (31) (Table 1; Figure 4). The number of E2 proteins in each vector is very similar, ranging from 29-36. Interestingly, there are slightly more E1s than E2s for *A. aegypti* and *C. quinquefasciatus*. We further compared the E2 proteins in *An. gambiae, Ae. aegypti, C. quinquefasciatus*, and *I. scapularis* (Figure S3). 

### Ubiquitin ligases (E3)

#### RING domain E3 ligase

 HMM searches were performed on selected vectors and the total number of RING E3s was calculated: *I. scapularis* (151), *An.gambiae* (147), *Ae. aegypti* (161), *C. quinquefasciatus* (156), *P. humanus* (127) and *R. prolixus* (86) (Table 1; Figure 4). The number of RING E3s found in each protein dataset is significantly higher than any other component in the ubiquitin machinery. We annotated four Pfam domains associated with RING E3 ligases: zf-C3HC4, zf-Apc11, RINGv, and Rtf2. The zf-C3HC4 domain contains the amino acid motif pattern Cys_3_HisCys_4_. The zf-C3HC4 subcategory was the most common RING-type ligase within selected vectors: *I. scapularis* (100), *An. gambiae* (82), *Ae. aegypti* (84), *C. quinquefasciatus* (87), *P. humanus* (80) and *R. prolixus* (56) (Table S2-7). 

We report the number of zf-Apc11 proteins in *I. scapularis* (28), *An.gambiae* (42), *Ae. aegypti* (43), *C. quinquefasciatus* (47), *P. humanus* (31) and *R. prolixus* (19) (Table S2-7). RINGv proteins were observed in all six vectors: *I. scapularis* (17), *An. gambiae* (12), *Ae. aegypti* (22), *C. quinquefasciatus* (12), *P. humanus* (16) and *R. prolixus* (11) (Table S2-7). Rtf2 contains a C_2_HC_2_ motif and plays a role in DNA replication [40]. We analyzed the number of Rtf2 within each vector: *I. scapularis* (6), *An. gambiae* (11), *Ae. aegypti* (12), *C. quinquefasciatus* (10), *P. humanus* (6) and *R. prolixus* (8) (Table S2-7). It is important to note that some RING proteins contained two or more RING domains and were annotated based on the subcategory with the lowest E-value (Table S11).

 We further analyzed the RING E3s in *An. gambiae, Ae. aegypti, C. quinquefasciatus*, and *I. scapularis* by phylogeny estimation (Figure S4, S5). Because of computational limitations and the large number of RING proteins, we created a separate analysis for each of the four vectors. We also identified clusters related to each RING Pfam domain within the phylogeny (Figure S4, S5). 

#### HECT domain E3 ligase

 We analyzed the number of HECT E3s in *I. scapularis* (9), *An. gambiae* (17), *Ae. aegypti* (17), *C. quinquefasciatus* (23), *P. humanus* (14) and *R. prolixus* (16) (Table 1; Figure 4). The number of HECT E3s observed in each species is significantly lower than the number of RING ligases. We further examined the HECT E3s against other E3 ligases in *An. gambiae, Ae. aegypti, C. quinquefasciatus*, and *I. scapularis* by phylogeny estimation (Figure S6). 

#### Cullin

 Cullin proteins form a RING E3 in order to create a scaffold that facilitates the ligation of ubiquitin to the target substrate [41]. We calculated the number of proteins associated with Cullin in *I. scapularis* (5), *An.gambiae* (6), *Ae. aegypti* (9), *C. quinquefasciatus* (4), *P. humanus* (7) and *R. prolixus* (8) (Table 1; Figure 4). The number of Cullin proteins is the smallest amongst the subcategories of ubiquitin ligases within each vector. This pattern is consistent with model species: *S. cerevisiae* (4), *D. melanogaster* (14), *H. sapiens* (16), and M. musculus (11) (Table 1). We then compared the Cullin proteins within *An. gambiae, Ae. aegypti, C. quinquefasciatus*, and *I. scapularis* against other E3 ligases (Figure S6). Similar to HECT ligases, most of the Cullin protein clusters were separated in the estimated tree. 

#### U-box Ubiquitin Ligase

 In addition to RING, HECT, and Cullin, recent studies have identified proteins that share many similarities with the RING finger, but lack the metal-chelating residue and many signature Cys residues in the original RING domain [42]. This protein family has been classified as the U-box, although it has previously been identified as E4 [43]. Here, we report the number of U-box ubiquitin ligases in *I. scapularis* (8), *An.gambiae* (10), *Ae. aegypti* (10), *C. quinquefasciatus* (12), *P. humanus* (7) and *R. prolixus* (8) (Table 1). Much like Cullin, U-box proteins account for only a small ratio of the total E3 ligases in each vector. When comparing U-boxes with other E3 ligases, we observed only two clusters of U-box proteins within the estimated tree (Figure S6). 

#### F-box

 The F-box domain is categorized by a structural motif that contains approximately 50 amino acids [44]. We used hmmsearch to reveal the following numbers of F-box proteins in the various disease vectors: *I. scapularis* (43), *An. gambiae* (39), *Ae. aegypti* (49), *C. quinquefasciatus* (151), *P. humanus* (32) and *R. prolixus* (39) (Table 2; Figure 4). The number of proteins associated with F-box is the second largest component for five out of six arthropod vectors. In model species, we observed a similar increase in the number of F-box and most other ubiquitination components: *S. cerevisiae* (12), *D. melanogaster* (46), *H. sapiens* (111), and M. musculus (104) (Table 1). We then extracted the sequences of F-box proteins in *An. gambiae, Ae. aegypti, C. quinquefasciatus*, and *I. scapularis* and estimated phylogenies for each species (Figure S7, S8). 

### Deubiquitinases (DUBs)

#### Ovarian tumor domain (OTU)

**Figure 5:**
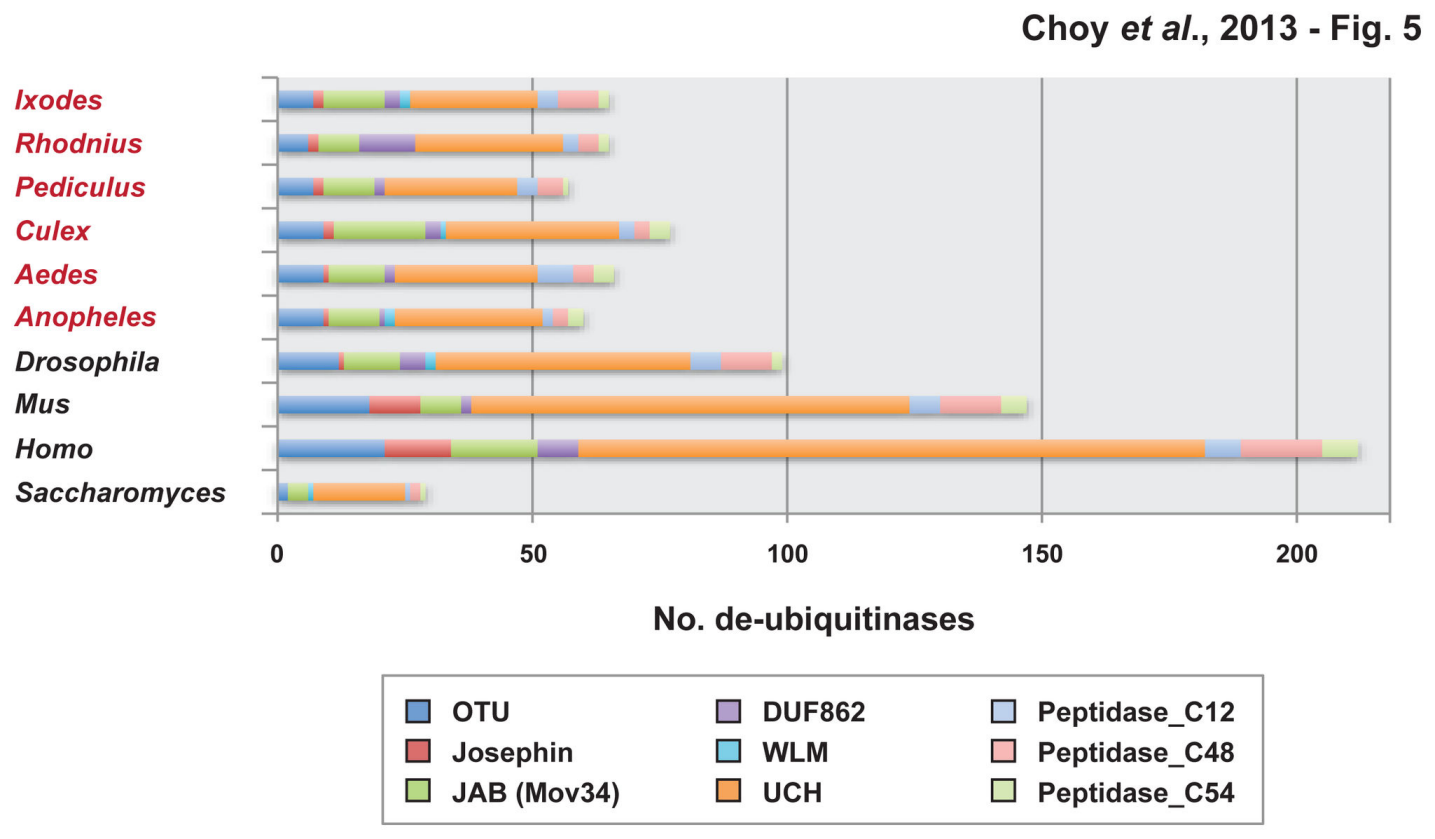
Composition of de-ubiquitinases within the genomes of four model eukaryotes and six medically important arthropod vectors. Using HMMER v.3.0, complete sets of proteins for all ten genomes were scanned for the presence of nine Pfam domain models for de-ubiquitinases: OTU (PF02338), Josephin (PF02099), JAB (PF01398), DUF862 (PF05903), WLM (PF08325), UCH (PF00443), Peptidase_C12 (PF01088), Peptidase_C48 (PF02902), and Peptidase_C54 (PF03416). Only protein matches to Pfam models with E-values <0.05 were selected for compilations.

 OTU DUBs have been known to hydrolyze K48, K63, and polyubiquitin chains [45]. Our study uncovered the number of OTU-associated proteins in the six selected vectors and four model organisms as: *I. scapularis* (7), *An. gambiae* (9), *Ae. aegypti* (9), *C. quinquefasciatus* (9), *P. humanus* (7), *R. prolixus* (6), *S. cerevisiae* (2), *D. melanogaster* (12), *H. sapiens* (21), and M. musculus (18) (Table 1; Figure 5). In most species, OTU-associated proteins were the third most common DUBs, accounting for approximately 12.5% of the total number of DUBs. Interestingly, the lowest ratio of OTU to DUBs was found in *H. sapiens*, where OTU accounted for only 9.9%. We further analyzed relatedness between OTU and other DUBs through estimated phylogenies across proteins from *An. gambiae, Ae. aegypti, C. quinquefasciatus*, and *I. scapularis* (Figure S9, S10). 

#### Josephin

 The Josephin domain is a highly conserved catalytic motif containing approximately 180 residues and was named after Machado-Joseph disease (MJD), a neurodegenerative condition [46]. We reported the number of Josephin-associated proteins in *I. scapularis* (2), *An. gambiae* (1), *Ae. aegypti* (1), *C. quinquefasciatus* (2), *P. humanus* (2), *R. prolixus* (2), *S. cerevisiae* (0), *D. melanogaster* (1), *H. sapiens* (13), and M. musculus (10) (Table 1; Figure 5). All selected vectors carried a very limited number of proteins containing the Josephin domain, ranging from 1-2. Interestingly, *H. sapiens* and *M. musculus* contained a significantly larger number of Josephin-related proteins. We then analyzed Josephin-associated DUBs against other DUBs for *An. gambiae, Ae. aegypti, C. quinquefasciatus*, and *I. scapularis* (Figure S9, S10). 

#### Jun activation domain-binding protein (JAB)

 The JAB domain is a component in a group of metalloproteases known as Jab1/MPN metalloenzyme (JAMM) DUBs [47]. This specific type of DUBs targets K63 polyubiquitin chains [48]. We analyzed the number of JAB-associated DUBs found within selected disease vectors: *I. scapularis* (12), *An. gambiae* (10), *Ae. aegypti* (11), *C. quinquefasciatus* (18), *P. humanus* (10) and *R. prolixus* (8) (Table 1; Figure 5). Proteins containing the JAB domain accounted for the second most abundant DUBs. Interestingly, the ratio of JAB-associated DUBs to total DUBs was much lower in *H. sapiens* and *M. musculus* than in the six vectors. Furthermore, the total number of proteins containing JAB found in *M. musculus* was smaller than any of those found in the analyzed vectors. We then compared JAB-containing DUBs with other DUBs in *An. gambiae, Ae. aegypti, C. quinquefasciatus*, and *I. scapularis* (Figure S9, S10). 

#### Domain of Unknown Function *862* (DUF862)

 We identified the number of DUF862-containing DUBs within *I. scapularis* (3), *An.gambiae* (1), *Ae. aegypti* (2), *C. quinquefasciatus* (3), *P. humanus* (2) and *R. prolixus* (11) (Table 1; Figure 5). The number of proteins displaying the DUF862 domain was relatively small. The lone exception was *R. prolixus*, where DUF862 DUBs account for the second highest number of DUBs. We then compared the DUF862-containing proteins with other DUBs in *An. gambiae, Ae. aegypti, C. quinquefasciatus*, and *I. scapularis* by constructing estimated phylogenies (Figure S9, S10). 

#### Wss1p‐like metalloproteases (WLM)

 The WLM domain is the defining characteristic of a family within Zn-dependent peptidases, termed the WLM DUBs. Although its functions are not fully elucidated, WLM DUBs have been associated with de-SUMOylation [49]. WLM was the rarest DUB found within all six vectors: *I. scapularis* (2), *An. gambiae* (2), *Ae. aegypti* (0)*, C. quinquefasciatus* (1), *P. humanus* (0), and *R. prolixus* (0) (Table 1; Figure 5). 

#### Ubiquitin carboxyl-terminal hydrolases (UCH)

 DUBs containing the UCH domain have been known to release ubiquitin in polyubiquitin chains by hydrolyzing its C-terminus [50]. We performed an hmmsearch for the total numbers of UCH DUBs within *I. scapularis* (25), *An. gambiae* (29), *Ae. aegypti* (28), *C. quinquefasciatus* (34), *P. humanus* (26) and *R. prolixus* (29) (Table 1; Figure 5). UCH-associated proteins account for the largest number of DUBs within every vector. The average ratio between UCH-containing proteins to total DUBs for all six vectors was approximately 41%, with the lowest being *I. scapularis* at 38%. In the estimated trees comparing UCH DUBs with other DUBs, we observed several UCH clusters for each vector: *An. gambiae* (6), *Ae. aegypti* (6), *C. quinquefasciatus* (8), and *I. scapularis* (5). (Figure S9, S10). 

#### Peptidase_C12, Peptidase_C48, Peptidase_C54 domains

Peptidase_C12 has been linked to the UCH family of DUBs, while the Peptidase_C48 domain is a C-terminal catalytic domain associated with the Ubl-specific protease 1 (ULP1) protease family [51,52]. Peptidase_C54 has not been previously studied, although our analysis has identified it as a domain similar to a DUF related to URM1 proteases [53]. We analyzed the number to DUBs containing each of the three peptidase domains: *I. scapularis* (Peptidase_C12: 4, Peptidase_C48: 8, Peptidase_C54: 2)*, An. gambiae* (Peptidase_C12: 2, Peptidase_C48: 3, Peptidase_C54: 3)*, Ae. aegypti* (Peptidase_C12: 7, Peptidase_C48: 4, Peptidase_C54: 4)*, C. quinquefasciatus* (Peptidase_C12: 3, Peptidase_C48: 3, Peptidase_C54: 4)*, P. humanus* (Peptidase_C12: 4, Peptidase_C48: 5, Peptidase_C54: 1), *R. prolixus* (Peptidase_C12: 3, Peptidase_C48: 4, Peptidase_C54: 2), *S. cerevisiae* (Peptidase_C12: 1, Peptidase_C48: 2, Peptidase_C54: 1), *D. melanogaster* (Peptidase_C12: 6, Peptidase_C48: 10, Peptidase_C54: 2), *H. sapiens* (Peptidase_C12: 7, Peptidase_C48: 16, Peptidase_C54: 7), and M. musculus (Peptidase_C12: 6, Peptidase_C48: 12, Peptidase_C54: 5) (Table 1; Figure 5). In most analyzed species, the number of Peptidase_C48 DUBs were the highest among the peptidase domain DUBs. The two exceptions were *Ae. aegypti*, where the number of Peptidase_C12-associated proteins was the highest, and *C. quinquefasciatus*, which contained one more deubiquitinase with a Peptidase_C54 domain. Each species possessed at least one DUB containing each of the peptidase domains. When analyzing the DUBs, we observed differences in the number of clusters formed for each vector (Figure S9, S10). 

## Conclusion

Vector-borne illnesses threaten public health in the tropics. Environmental changes due to globalization and the lack of effective vaccines are also contributing to the spread of these maladies to temperate climates. Thus, novel treatments are necessary in the clinic. One effective therapeutic strategy recently used to treat cancer, neurodegenerative disorders and some infectious diseases is pharmacological intervention targeting (de)ubiquitination. However, the use of (de)ubiquitination as a therapeutic target to combat arthropod-borne diseases has not been established. A possible reason for this gap in translational research is the inherent complexity of vector-borne diseases. Another possibility is the lack of communication among scientists. Pharmacologists and chemists do not typically interact with vector biologists, and vector biologists have little incentive to interact with the drug development community. 

Here we took advantage of publicly available arthropod genomes and described the ubiquitin machinery of *An. gambiae, Ae. aegypti, C. quinquefasciatus, I. scapularis, P. humanus* and *R. prolixus*. Although independent work will be necessary to validate our bioinformatics analysis, empirical evidence suggests that the ubiquitin machinery is present in disease vectors. Recently, Severo et al., 2013 characterized a RING-type E3 ubiquitin ligase named x-linked inhibitor of apoptosis protein (XIAP) and showed the importance of ubiquitination for microbial pathogenesis in ticks [54]. Similarly, targeting of genes by RNAi from the ubiquitin-mediated pathway affected bacterial infection in arthropod vectors [55]. Corroborating with these findings, Huang and colleagues demonstrated that monoubiquitinated proteins decorate pathogen-occupied vacuolar membranes during infection of embryonic tick cells [56]. In summary, this report should: (1) provide a framework for studying ubiquitination in disease vectors; (2) generate a basis for empirical experimentation correlating arthropod physiology and disease; and (3) potentially unveil novel pharmacological targets to interfere with vector-borne diseases. 

## Supporting Information

Table S1 **Source and release date of protein datasets.**
(PDF)

Table S2 **List of proteins in *An*.** *gambiae* identified by the HMM search.
(XLSX)

Table S3 **List of proteins in *Ae. aegypti* identified by the HMM search.**
(XLSX)

Table S4 **List of proteins in *C. quinquefasciatus* identified by the HMM search.**
(XLSX)

Table S5 **List of proteins in *I. scapularis* identified by the HMM search.**
(XLSX)

Table S6 **List of proteins in *P. humanus* identified by the HMM search.**
(XLSX)

Table S7 **List of proteins in *R. prolixus* identified by the HMM search.**
(XLSX)

Table S8 **List of proteins in *D. melanogaster* identified by the HMM search.**
(XLSX)

Table S9 **List of proteins in *H. sapiens* identified by the HMM search.**
(XLSX)

Table S10 **List of proteins in *M. musculus* identified by the HMM search.**
(XLSX)

Table S11 **List of protein identifications containing sequences matching two or more Pfam domains.**
(XLSX)

Figure S1 **Ubiquitin and ubiquitin-like proteins in *I. scapularis, An*.** ***gambiae, Ae. aegypti*, and *C. quinquefasciatus***. Protein sequences matched with ubiquitin and ubiquitin-like proteins were aligned using MUSCLE and a phylogeny was estimated with the PhyML software. Sequences with a bootstrap value lower than 50 were manually removed. Bootstrap values ranged from 0.64 - 0.86 for highlighted clustered categories (SUMO, ATG8, UB S30, NEDD8, HUB1, URM1).
(TIF)

Figure S2 **Ubiquitin and ubiquitin-like activating enzymes in *I. scapularis, An*.** ***gambiae, Ae. aegypti*, and *C. quinquefasciatus***. Phylogeny of ubiquitin and ubiquitin-like activating enzymes. Matched sequences for ubiquitin and ubiquitin-like activating enzymes were aligned using MUSCLE and a phylogeny was estimated with the PhyML software. Bootstrap values ranged from 0.43 - 0.83 for highlighted clustered categories (UBA1, UBA2, UBA3, ATG7, and UBA1-like).
(TIF)

Figure S3 **Ubiquitin and ubiquitin-like conjugating enzymes in *I. scapularis, An*.** ***gambiae, Ae. aegypti*, and *C. quinquefasciatus***. Protein sequences matched with ubiquitin and ubiquitin-like conjugating enzymes were aligned using MUSCLE and a phylogeny was estimated with the PhyML software.
(TIF)

Figure S4 **Phylogeny of RING and RING-like ubiquitin ligases in *An*.** ***gambiae and Ae. aegypti***. Protein sequences matched with ubiquitin and ubiquitin-like ligases were aligned using MUSCLE and a phylogeny was estimated with the PhyML software. Proteins not listed under a specific subset were categorized as zf-C3HC4. Phylogeny of RING and RING-like ubiquitin ligases in (A) *An. gambiae* and (B) *Ae. aegypti*. Bootstrap values ranged from (A) 0.41 - 0.80 and (B) 0.41 - 0.83 for highlighted clustered categories (RINGv, Rtf2, zf-Apc11).
(TIF)

Figure S5 **Phylogeny of RING and RING-like ubiquitin ligases in *C. quinquefasciatus* and *I. scapularis*.** Protein sequences matched with ubiquitin and ubiquitin-like ligases were aligned using MUSCLE and a phylogeny was estimated with the PhyML software. Proteins not listed under a specific subset were categorized into the zf-C3HC4 classification. Phylogeny of RING and RING-like ubiquitin ligases in (A) *C. quinquefasciatus* and (B) *I. scapularis*. Bootstrap values ranged from (A) 0.43 - 0.83 and (B) 0.46 - 0.83 for highlighted clustered categories (RINGv, Rtf2, zf-Apc11).
(TIF)

Figure S6 **HECT, Cullin and U-box ligases in *I. scapularis, An*.** ***gambiae, Ae. aegypti*, and *C. quinquefasciatus***. Protein sequences matched with ubiquitin and ubiquitin-like ligases were aligned using MUSCLE and the phylogeny was estimated with the PhyML software. Sequences with bootstrap values lower than 50 were manually removed. Bootstrap values ranged from 0.35 - 0.88 for highlighted clustered categories (HECT, Cullin, U-Box).
(TIF)

Figure S7 **Phylogenetic trees of F-box ubiquitin ligases in *An*.** ***gambiae and Ae. aegypti***. Protein sequences matched with F-box ligases were aligned using MUSCLE and the phylogeny was estimated with the PhyML software. Sequences with a bootstrap values lower than 50 were manually removed. Phylogenetic trees of F-box ubiquitin ligases in (A) *An. gambiae* and (B) *Ae. aegypti*.
(TIF)

Figure S8 **Phylogenetic trees of F-box ubiquitin ligases in *C. quinquefasciatus* and *I. scapularis*.** Protein sequences matched with F-box ligases were aligned using MUSCLE and the phylogeny was estimated with the PhyML software. Sequences with a bootstrap values lower than 50 were manually removed. Phylogenetic trees of F-box ubiquitin ligases in (A) *C. quinquefasciatus* and (B) *I. scapularis*.
(TIF)

Figure S9 **Phylogenetic trees of deubiquitinases in *An*.** ***gambiae and Ae. aegypti***. Protein sequences were aligned using MUSCLE and a phylogeny was estimated using the maximum likelihood method. Phylogeny of deubiquitinases in (A) *An. gambiae* and (B) *Ae. aegypti*. Bootstrap values ranged from (A) 0.43 - 0.87 and (B) 0.37 - 0.84 for highlighted clustered categories (UCH, OTU, WLM, JAB, Peptidase_C12, Peptidase_C54, Peptidase_C48, Josephin and DUF862).
(TIF)

Figure S10 **Phylogenetic trees of deubiquitinases in *C. quinquefasciatus* and *I. scapularis*.** Protein sequences were aligned using MUSCLE and a phylogeny was estimated using the maximum likelihood method. Phylogeny of deubiquitinases in (A) *C. quinquefasciatus* and (B) *I. scapularis*. Bootstrap values ranged from (A) 0.39 - 0.83 and (B) 0.37 - 0.78 for highlighted clustered categories (UCH, OTU, WLM, JAB, Peptidase_C12, Peptidase_C54, Peptidase_C48, Josephin and DUF862).
(TIF)
