# Supplementary material for: Decoding the Ubiquitin-Mediated Pathway of Arthropod Disease Vectors
Source: PLoS One. 2013 Oct 21;8(10):e78077. doi: 10.1371/journal.pone.0078077 (PMC3804464; doi:10.1371/journal.pone.0078077)
Supplement: Table S1 — Source and release date of protein datasets. (PDF) [file pone.0078077.s001.pdf]

Table S1. Source and release date of proteome dataset

Organism	Source	Version [Release Date]
Anopheles gambiae	http://agambiae.vectorbase.org/downloads/public_data/organism_data/agambiae/Geneset/agambiae.PEPTIDES-AgamP3.6.fa.gz	3.6 [DEC 2010]
Aedes aegypti	http://aaegypti.vectorbase.org/downloads/public_data/organism_data/aaegypti/Geneset/aaegypti.PEPTIDES-AaegL1.3.fa.gz	1.3 [MAR 2012]
Culex quinquefasciatus	http://cquinquefasciatus.vectorbase.org/downloads/public_data/organism_data/cquinquefasciatus/Geneset/cquinquefasciatus.PEPTIDES-CpipJ1.3.fa.gz	1.3 [JAN 2012]
Ixodes scapularis	http://iscapularis.vectorbase.org/downloads/public_data/organism_data/iscapularis/Geneset/iscapularis.PEPTIDES-IscaW1.2.fa.gz	1.2 [MAR 2012]
Pediculus humanus	http://phumanus.vectorbase.org/downloads/public_data/organism_data/phumanus/Geneset/phumanus.PEPTIDES-PhumU1.2.fa.gz	1.2 [FEB 2010]
Rhodnius prolixus	http://rprolixus.vectorbase.org/downloads/public_data/organism_data/rprolixus/Geneset/rprolixus.PEPTIDES-RproC1.fa.gz	1.0[JUN 2010]
Saccharomyces cerevisiae	ftp://genome-ftp.stanford.edu/pub/yeast/data_download/sequence/genomic_sequence/orf_protein/orf_trans_all.fasta.gz	N/A [FEB 2012]
Drosophila melanogaster	ftp://ftp.flybase.net/genomes/Drosophila_melanogaster/dmel_r5.48_FB2012_06/fasta/dmel-all-translation-r5.48.fasta.gz	5.48[OCT 2012]
Mus musculus	ftp://ftp.ncbi.nlm.nih.gov/genomes/M_musculus/protein/protein.fa.gz	N/A [OCT 2012]
Homo sapiens	ftp://ftp.ncbi.nlm.nih.gov/genomes/H_sapiens/protein/protein.fa.gz	N/A [NOV 2012]
