# Supplementary figures and images for: Decoding the Ubiquitin-Mediated Pathway of Arthropod Disease Vectors
Source: PLoS One. 2013 Oct 21;8(10):e78077. doi: 10.1371/journal.pone.0078077 (PMC3804464; doi:10.1371/journal.pone.0078077)

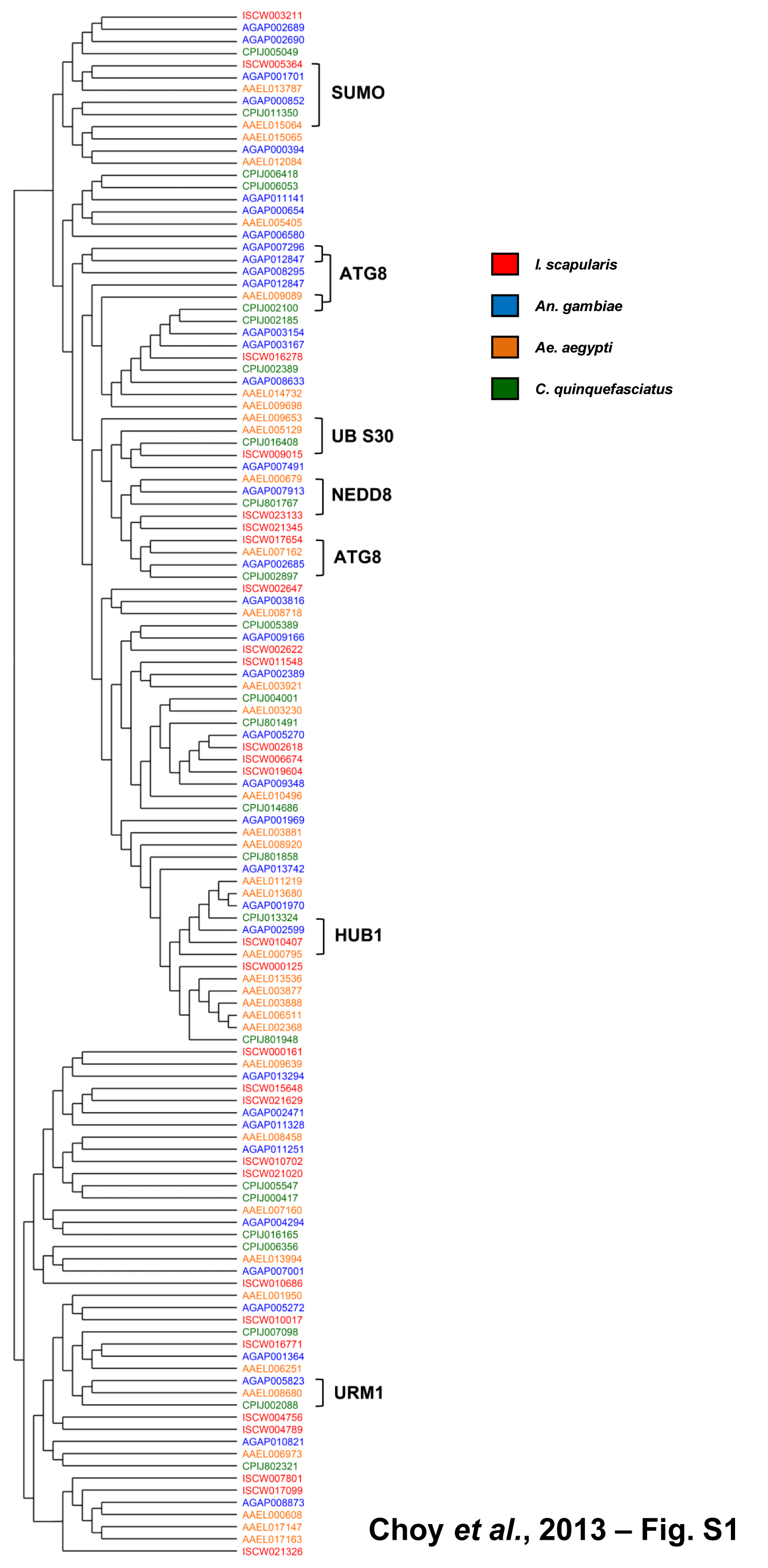

Supplement: Figure S1 — Ubiquitin and ubiquitin-like proteins in I. scapularis, An. gambiae, Ae. aegypti, and C. quinquefasciatus. Protein sequences matched with ubiquitin and ubiquitin-like proteins were aligned using MUSCLE and a phylogeny was estimated with the PhyML software. Sequences with a bootstrap value lower than 50 were manually removed. Bootstrap values ranged from 0.64 - 0.86 for highlighted clustered categories (SUMO, ATG8, UB S30, NEDD8, HUB1, URM1). (TIF) [file pone.0078077.s012.tif]

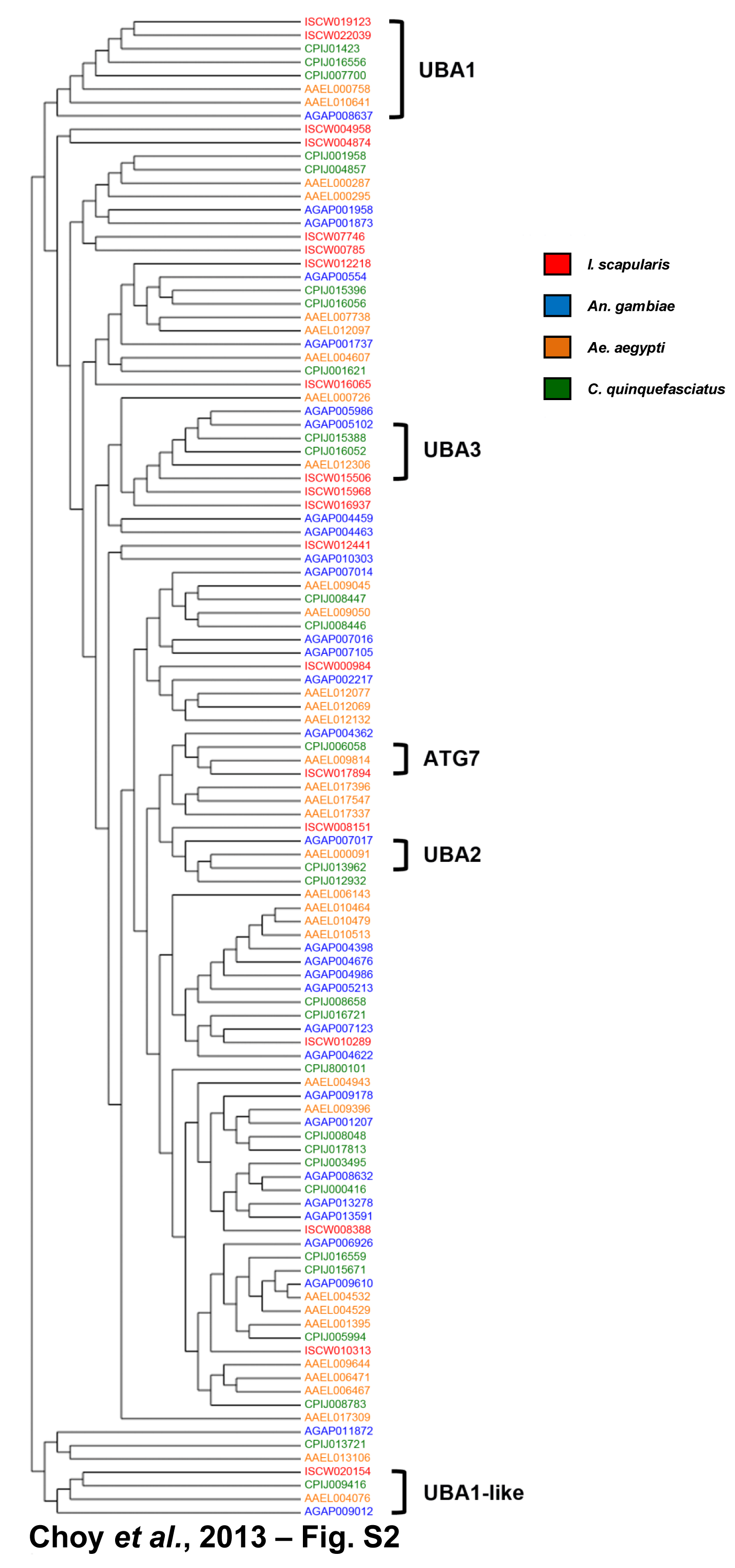

Supplement: Figure S2 — Ubiquitin and ubiquitin-like activating enzymes in I. scapularis, An. gambiae, Ae. aegypti, and C. quinquefasciatus. Phylogeny of ubiquitin and ubiquitin-like activating enzymes. Matched sequences for ubiquitin and ubiquitin-like activating enzymes were aligned using MUSCLE and a phylogeny was estimated with the PhyML software. Bootstrap values ranged from 0.43 - 0.83 for highlighted clustered categories (UBA1, UBA2, UBA3, ATG7, and UBA1-like). (TIF) [file pone.0078077.s013.tif]

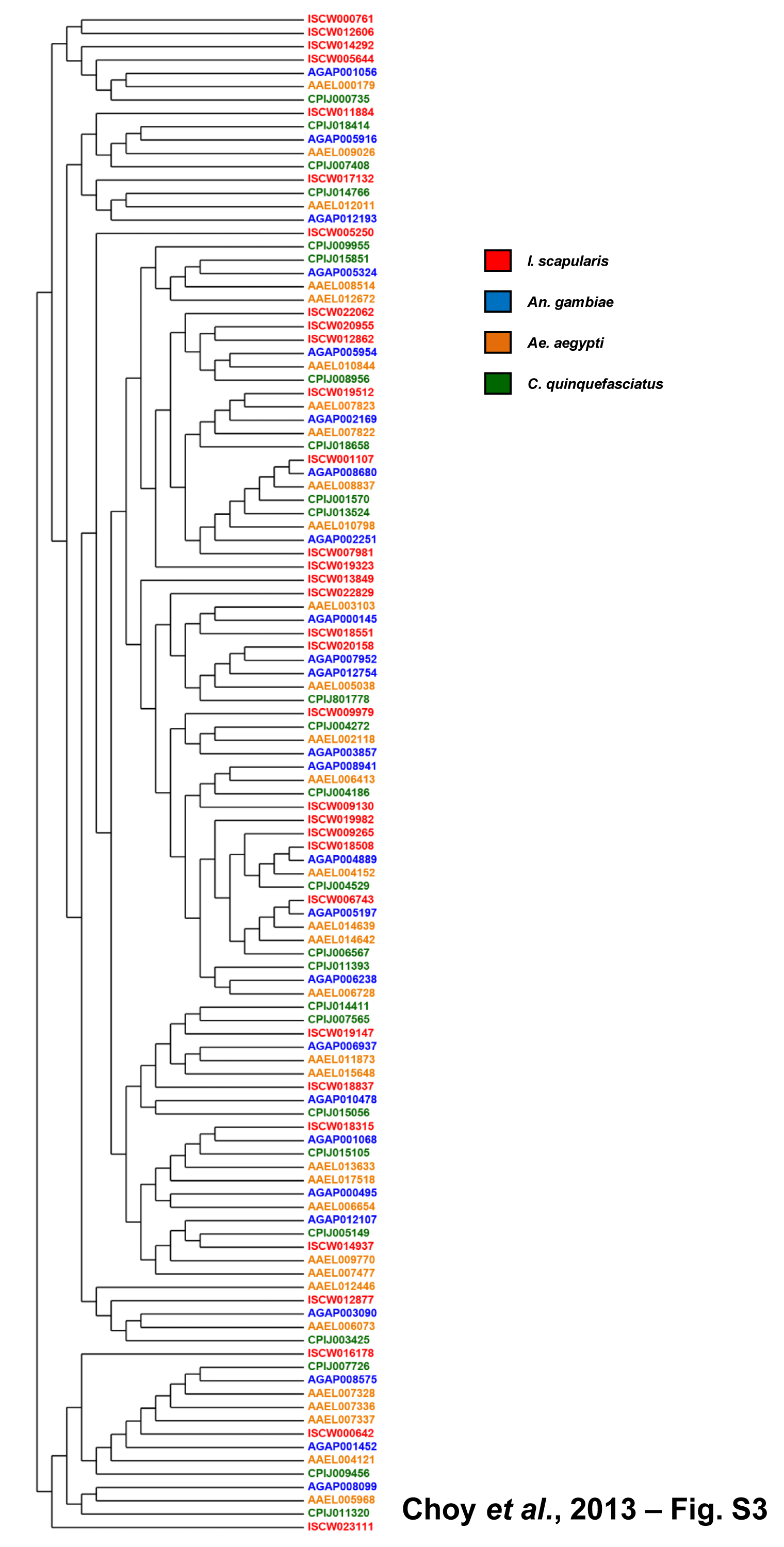

Supplement: Figure S3 — Ubiquitin and ubiquitin-like conjugating enzymes in I. scapularis, An. gambiae, Ae. aegypti, and C. quinquefasciatus. Protein sequences matched with ubiquitin and ubiquitin-like conjugating enzymes were aligned using MUSCLE and a phylogeny was estimated with the PhyML software. (TIF) [file pone.0078077.s014.tif]

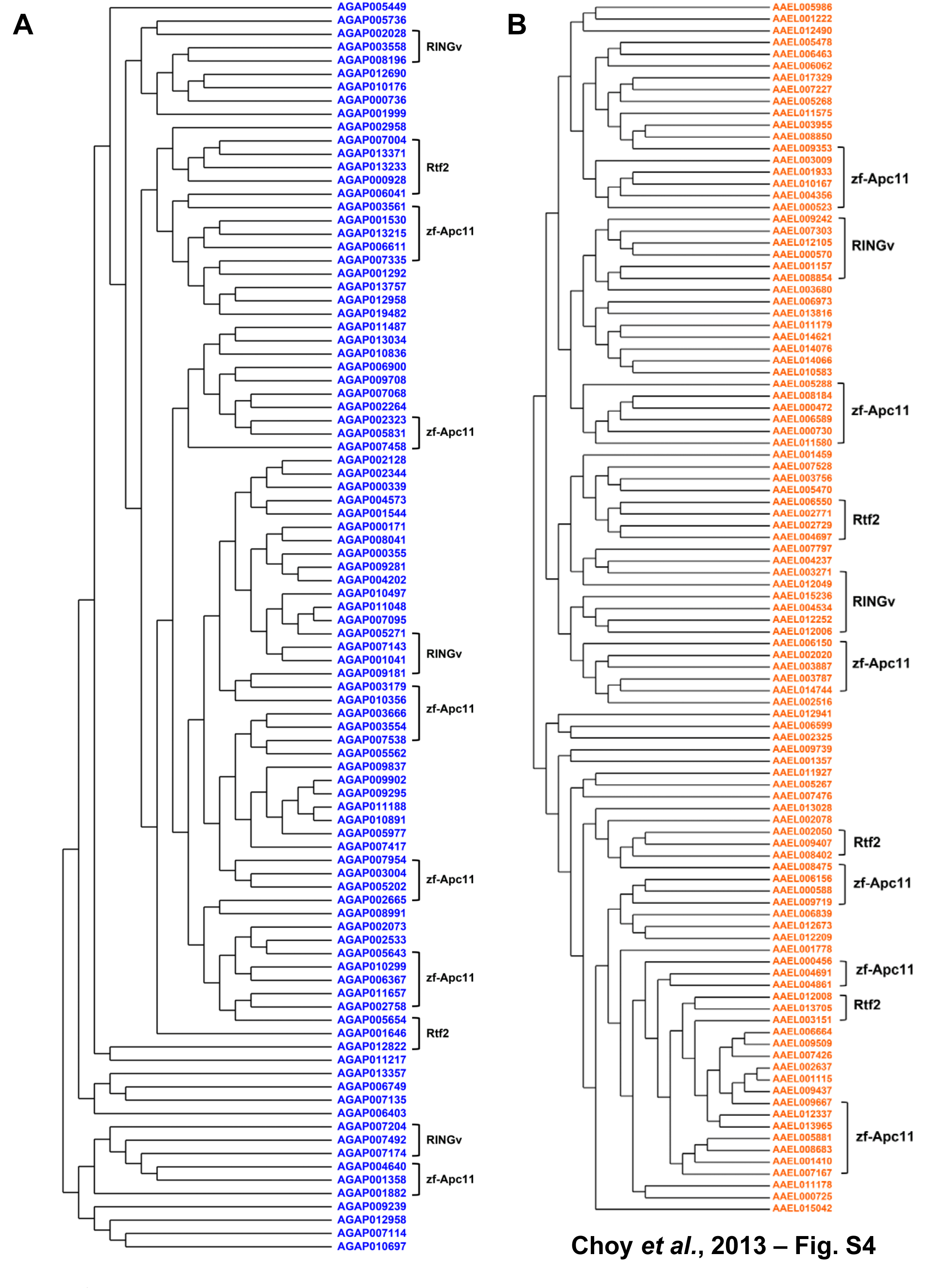

Supplement: Figure S4 — Phylogeny of RING and RING-like ubiquitin ligases in An. gambiae and Ae. aegypti. Protein sequences matched with ubiquitin and ubiquitin-like ligases were aligned using MUSCLE and a phylogeny was estimated with the PhyML software. Proteins not listed under a specific subset were categorized as zf-C3HC4. Phylogeny of RING and RING-like ubiquitin ligases in (A) An. gambiae and (B) Ae. aegypti. Bootstrap values ranged from (A) 0.41 - 0.80 and (B) 0.41 - 0.83 for highlighted clustered categories (RINGv, Rtf2, zf-Apc11). (TIF) [file pone.0078077.s015.tif]

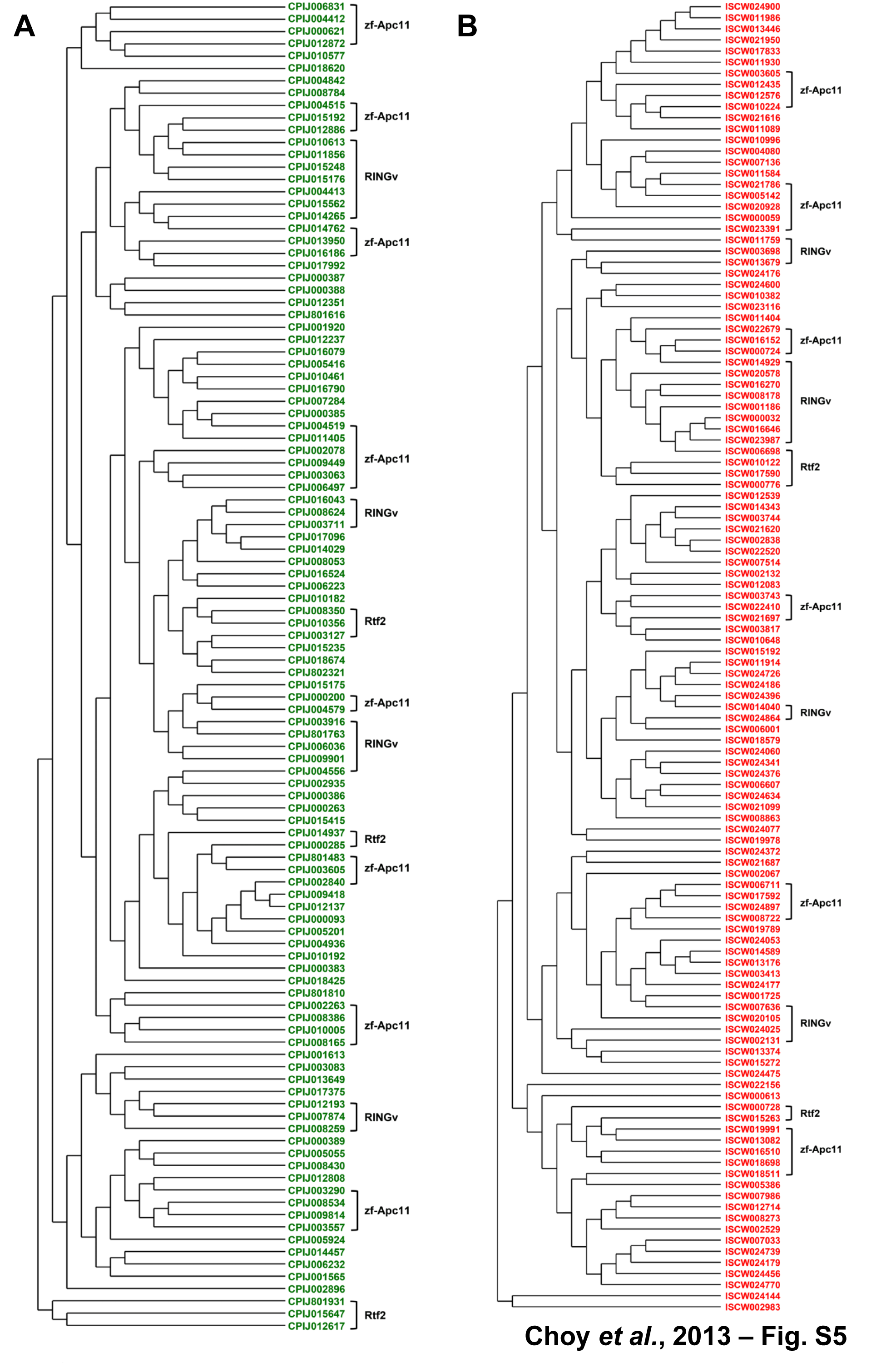

Supplement: Figure S5 — Phylogeny of RING and RING-like ubiquitin ligases in C. quinquefasciatus and I. scapularis. Protein sequences matched with ubiquitin and ubiquitin-like ligases were aligned using MUSCLE and a phylogeny was estimated with the PhyML software. Proteins not listed under a specific subset were categorized into the zf-C3HC4 classification. Phylogeny of RING and RING-like ubiquitin ligases in (A) C. quinquefasciatus and (B) I. scapularis. Bootstrap values ranged from (A) 0.43 - 0.83 and (B) 0.46 - 0.83 for highlighted clustered categories (RINGv, Rtf2, zf-Apc11). (TIF) [file pone.0078077.s016.tif]

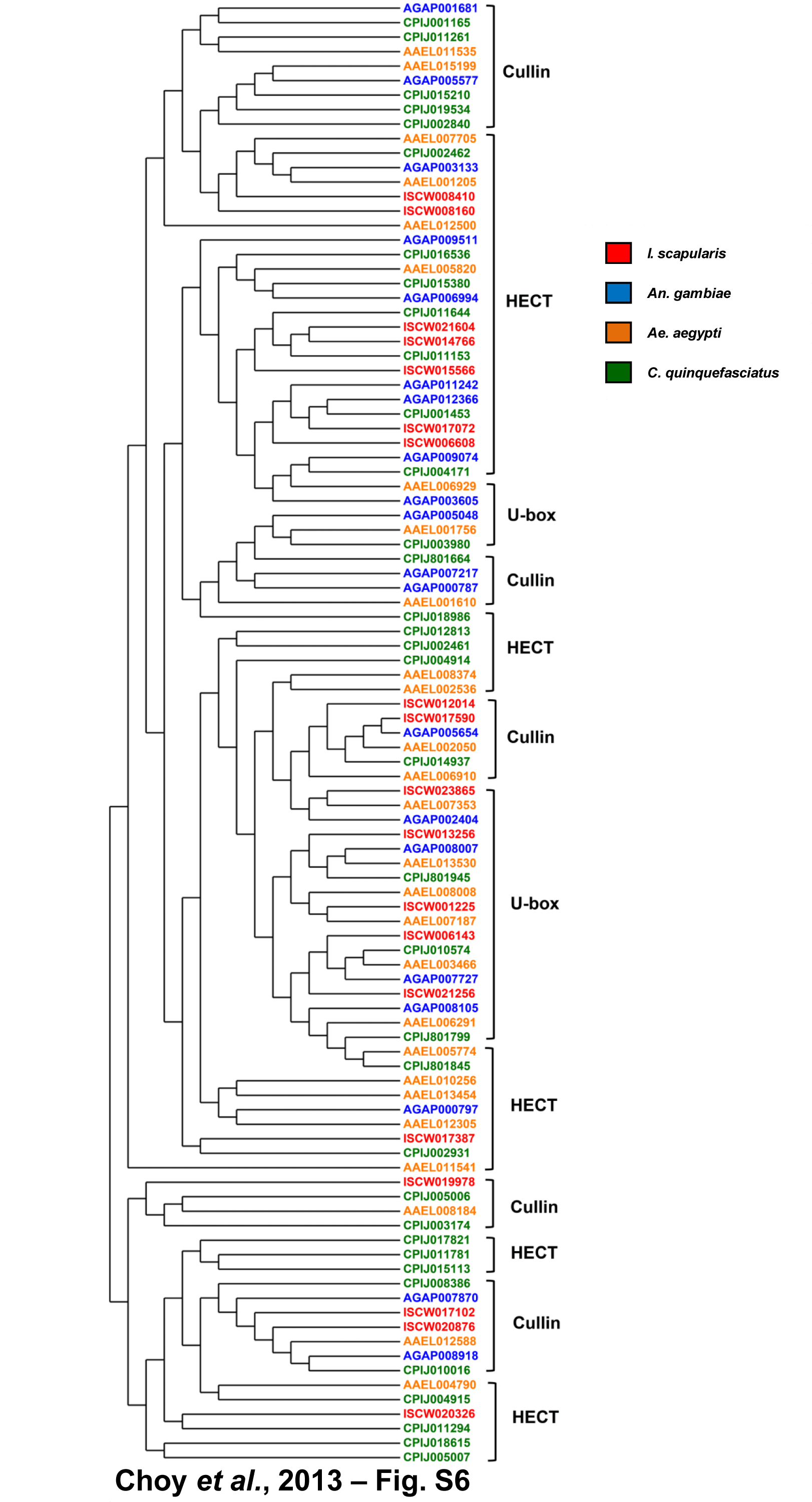

Supplement: Figure S6 — HECT, Cullin and U-box ligases in I. scapularis, An. gambiae, Ae. aegypti, and C. quinquefasciatus. Protein sequences matched with ubiquitin and ubiquitin-like ligases were aligned using MUSCLE and the phylogeny was estimated with the PhyML software. Sequences with bootstrap values lower than 50 were manually removed. Bootstrap values ranged from 0.35 - 0.88 for highlighted clustered categories (HECT, Cullin, U-Box). (TIF) [file pone.0078077.s017.tif]

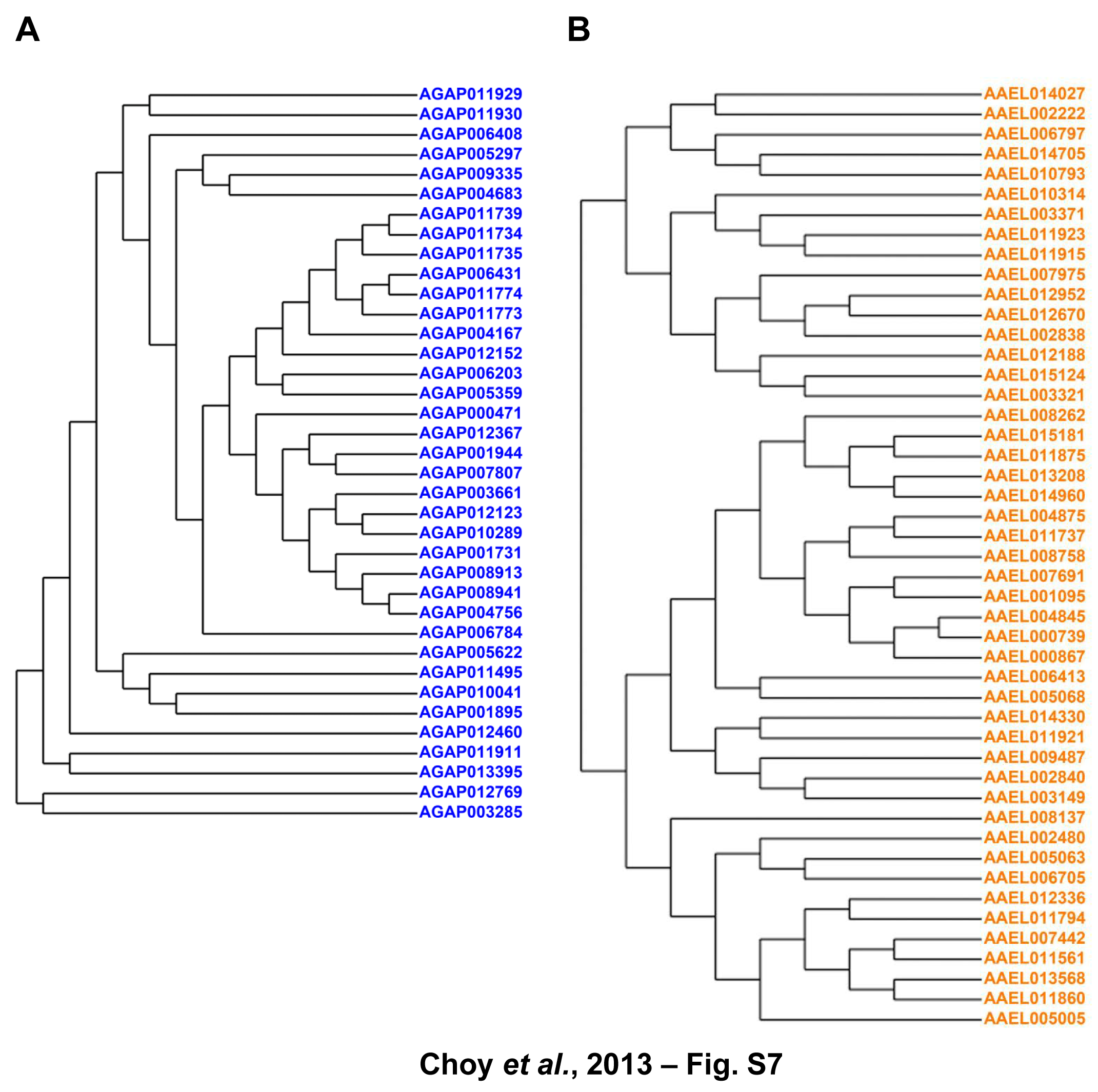

Supplement: Figure S7 — Phylogenetic trees of F-box ubiquitin ligases in An. gambiae and Ae. aegypti. Protein sequences matched with F-box ligases were aligned using MUSCLE and the phylogeny was estimated with the PhyML software. Sequences with a bootstrap values lower than 50 were manually removed. Phylogenetic trees of F-box ubiquitin ligases in (A) An. gambiae and (B) Ae. aegypti. (TIF) [file pone.0078077.s018.tif]

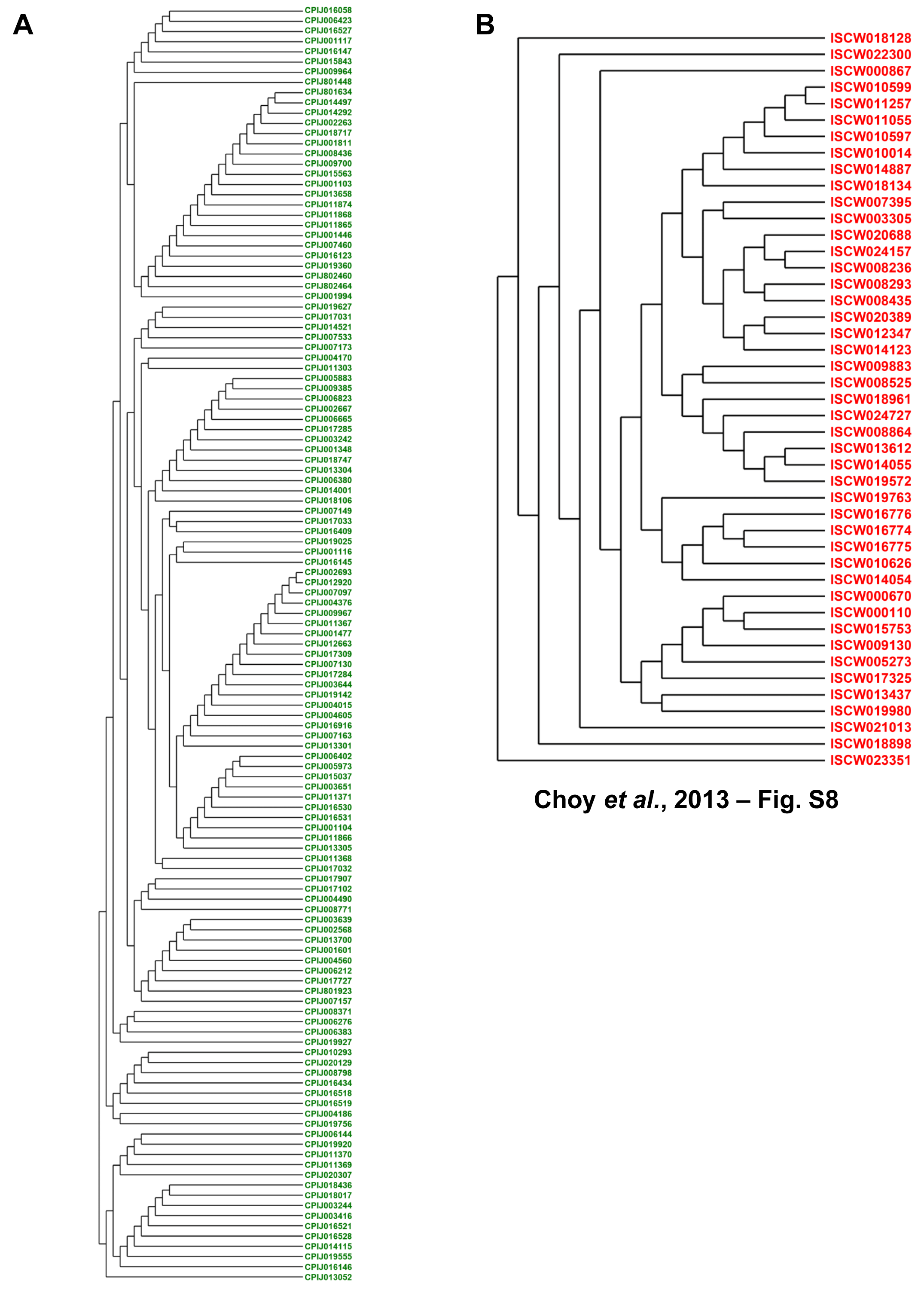

Supplement: Figure S8 — Phylogenetic trees of F-box ubiquitin ligases in C. quinquefasciatus and I. scapularis. Protein sequences matched with F-box ligases were aligned using MUSCLE and the phylogeny was estimated with the PhyML software. Sequences with a bootstrap values lower than 50 were manually removed. Phylogenetic trees of F-box ubiquitin ligases in (A) C. quinquefasciatus and (B) I. scapularis. (TIF) [file pone.0078077.s019.tif]

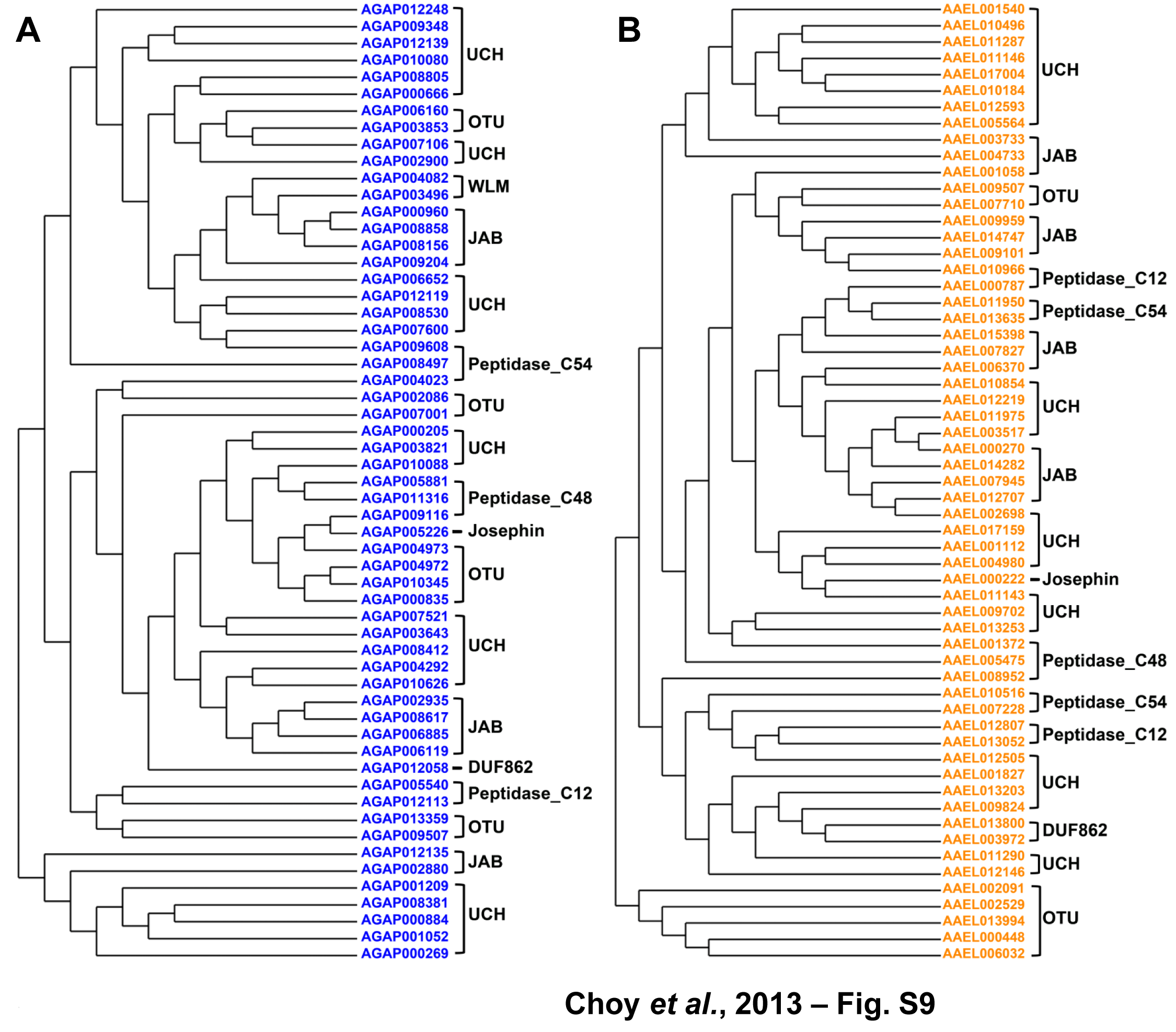

Supplement: Figure S9 — Phylogenetic trees of deubiquitinases in An. gambiae and Ae. aegypti. Protein sequences were aligned using MUSCLE and a phylogeny was estimated using the maximum likelihood method. Phylogeny of deubiquitinases in (A) An. gambiae and (B) Ae. aegypti. Bootstrap values ranged from (A) 0.43 - 0.87 and (B) 0.37 - 0.84 for highlighted clustered categories (UCH, OTU, WLM, JAB, Peptidase_C12, Peptidase_C54, Peptidase_C48, Josephin and DUF862). (TIF) [file pone.0078077.s020.tif]

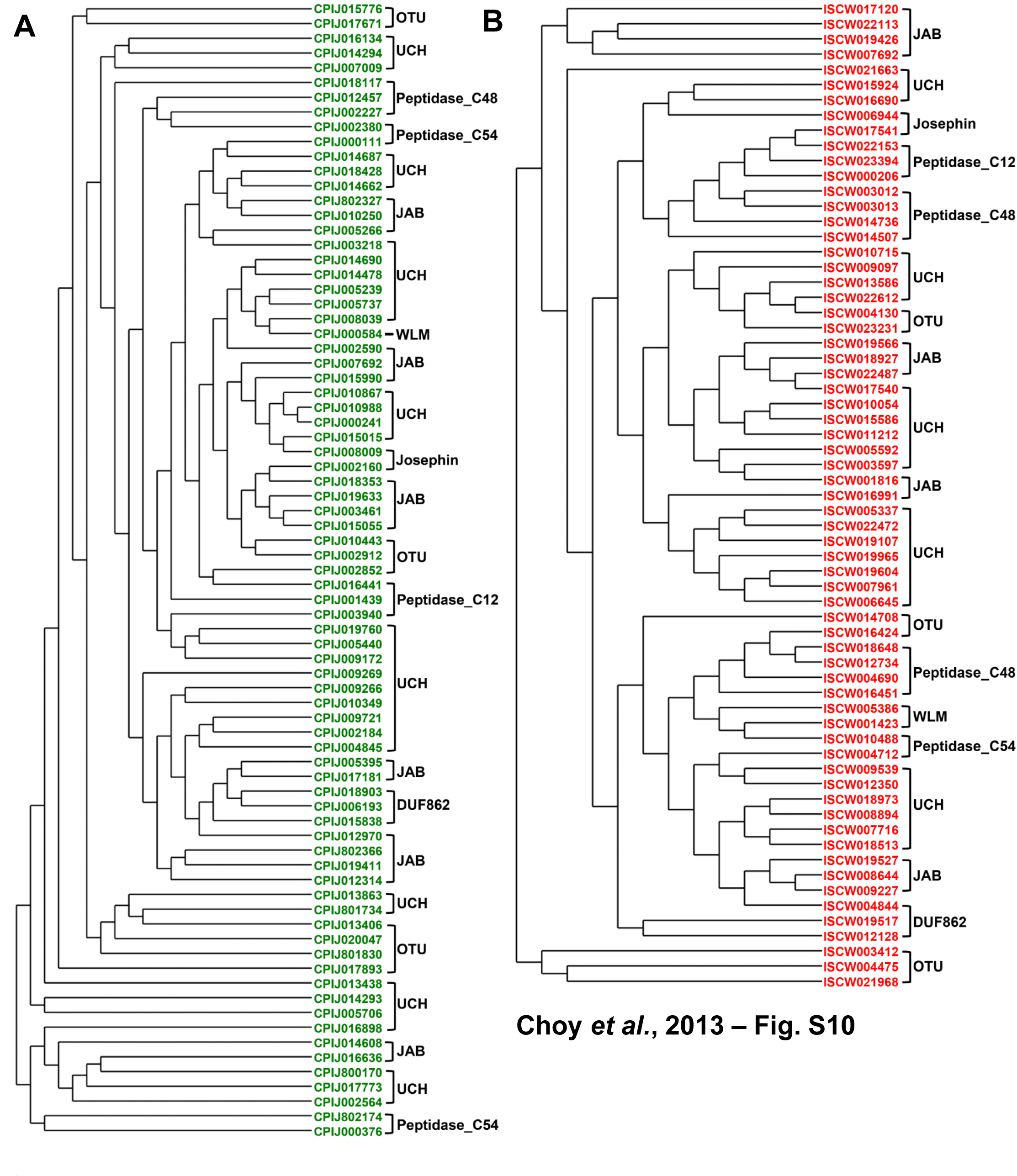

Supplement: Figure S10 — Phylogenetic trees of deubiquitinases in C. quinquefasciatus and I. scapularis. Protein sequences were aligned using MUSCLE and a phylogeny was estimated using the maximum likelihood method. Phylogeny of deubiquitinases in (A) C. quinquefasciatus and (B) I. scapularis. Bootstrap values ranged from (A) 0.39 - 0.83 and (B) 0.37 - 0.78 for highlighted clustered categories (UCH, OTU, WLM, JAB, Peptidase_C12, Peptidase_C54, Peptidase_C48, Josephin and DUF862). (TIF) [file pone.0078077.s021.tif]
